# A Needle in the Haystack: A Rare Case of Spontaneous Tumor Lysis in Newly Diagnosed Chronic Lymphocytic Leukemia Unmasked by Acute Renal Failure

**DOI:** 10.7759/cureus.11279

**Published:** 2020-10-31

**Authors:** Milap Desai, Meghana Parsi, Rashmika Potdar, Rashmi Sanjay

**Affiliations:** 1 Hematology/Oncology, Drexel University College of Medicine, Philadelphia, USA; 2 Internal Medicine, Crozer-Keystone Health System, Upland, USA; 3 Hematology and Oncology, Crozer-Keystone Health System, Upland, USA; 4 Hematology and Oncology, Crozer Keystone Health System, Upland, USA

**Keywords:** tumor lysis syndrome, spontaneous tumor lysis syndrome, benign prostatic hyperplasia, smudge cells, chronic lymphocytic leukemia

## Abstract

Tumor lysis syndrome (TLS) is the phenomenon of metabolic derangements that typically follows the initiation of cytotoxic chemotherapy. Metabolic disturbances include hyperphosphatemia, hyperkalemia, hyperuricemia and hypocalcemia. Hematological malignancies are associated with spontaneous TLS (STLS), which is cell lysis in the absence of chemotherapy. STLS is extremely rare in chronic lymphocytic leukemia (CLL). This has been documented only once in the medical literature, making this an extraordinarily uncommon case. We present here a 68-year-old male with a history of benign prostatic hyperplasia (BPH) who is admitted for a two-week history of abdominal pain and three days of anuria, despite adequate fluid intake. Laboratory values yielded a greatly elevated leukocyte count with a lymphocytic predominance and smudge cells. Potassium, phosphorus, and uric acid were also significantly increased. EKG revealed peaked T-waves. Flow cytometry confirmed the presence of an abnormal B-cell population consistent with B-cell chronic lymphocytic leukemia, with the following markers: CD19+, CD20+, CD23+, CD5+, CD10-. He was diagnosed with CLL and treated with aggressive fluid resuscitation, allopurinol and rasburicase. The patient had another similar episode within one month. His CLL fluorescence in-situ hybridization (FISH) showed complex cytogenetics with unmutated IgVH and he was subsequently started on ibrutinib.

## Introduction

Chronic lymphocytic leukemia (CLL) is a hematological neoplasm of mature B cells characterized by monoclonal B lymphocytes. CLL is the most common leukemia among adults in the western countries, with an incidence of 25%-30% [1]. CLL is more common in men, male to female ratio of 1.3:1 to 1.7:1, and the median age is 70 upon diagnosis [1-3]. This affliction tends to be seen more in Caucasian adults than African-American or Asian Pacific Islander counterparts [2,4]. Interestingly, CLL is thought to be explained mostly by genetic factors [5,6].

Symptomology of CLL is highly variable ranging from none to lymphadenopathy to classic “B” symptoms, which includes unintentional weight loss ≥10 pounds in the previous six months, fevers greater than 100.4 F for >2 weeks without evidence of other causes, and drenching night sweats (48). Splenomegaly can be seen in up to 25%-55% of cases [7,8]. CLL can also involve other organs such as liver, skin, and gastrointestinal mucosal tissue [7-10]. Treatment for CLL is dependent on the patient’s signs and symptoms and is only recommended in “active” disease resulting in critical end-organ damage, worsening thrombocytopenia and anemia, lymph nodes exceeding 10 cm in diameter, symptomatic lymph node enlargements, symptomatic or massive splenomegaly “B” symptoms like weight loss, night sweats, and fevers.

A prominent lymphocytosis is typically seen in CLL. Absolute lymphocyte count of >5000/microliters is used as a threshold when diagnosing CLL; however, many patients present with up to or greater than 100,000/microliters of B-cells. Moreover, neutropenia, anemia, and thrombocytopenia have been documented alongside a greatly elevated absolute lymphocyte count. Also, approximately 25% of CLL patients present with hypogammaglobulinemia at the time of diagnosis, exposing the patient to an increased risk of major infections [11].

A majority of hematological cancer has been linked to tumor lysis syndrome (TLS). TLS is predominantly observed in the setting of quick cell turnover, great tumor burden, initiation of cytotoxic agents or kidney failure. Rapid lysis of tumor cells releases intracellular content such as uric acid, phosphate, and potassium into the systemic circulation causing serious downstream effects. The Cairo-Bishop criteria for TLS diagnosis are outlined in Table 2 [12].

Hyperuricemia due to catabolism of released nucleic acids eventually crystallizes in the acidic environment of the distal tubules. Precipitation and deposition of uric acid crystals can cause new-onset acute kidney injury or exacerbate ongoing kidney failure, worsening the severity of TLS. Patients classified as high-risk or intermediate-risk of developing TLS are often pretreated with uric acid lowering agents, such as allopurinol and rasburicase. Prophylaxis with these drugs decreases uric-acid mediated complications.

Hyperphosphatemia following the lysis of tumor cells also causes organ damage via the precipitation and deposition of calcium phosphate crystals in the kidney. High phosphate levels in the blood cause hypocalcemia through various physiological mechanisms. Calcium-phosphate crystals can deposit in the heart producing arrhythmias. Hyperkalemia is another metabolic derangement seen in TLS that presents a high risk of arrhythmias.

Spontaneous tumor lysis syndrome (STLS) describes the phenomenon of tumor lysis in the absence of a deliberate trigger such as administration of cytotoxic agents or glucocorticoid therapy. STLS has been linked to various aggressive hematological malignancies such as acute leukemias and non-Hodgkin’s lymphoma. Estimates of STLS prevalence have not been confirmed, however it is thought to be rare. STLS in CLL is exceedingly rare, only once before documented in medical literature. We present here a case of STLS and acute kidney failure in a male patient, with no prior cancer history.

## Case presentation

A 68-year-old man with a history of hypertension, hyperlipidemia, type II diabetes, benign prostatic hyperplasia (BPH), presented from home with two weeks of abdominal pain, diarrhea, and decreased urine output. Upon further investigation in the emergency department, the patient mentioned he has not made urine in over three days although he has been consuming water regularly. He noted a “dull”, aching pain in his abdomen with feelings of fullness and distension. He denied weight loss, fevers, chills, chest pain, or shortness of breath. 

In the emergency room, the patient was saturating at 88% on room air and recorded a blood pressure of 112/43 mm Hg (right). Other vital signs showed: pulse 87, respiratory rate 18, temperature 98.2F. Physical exam was notable for mild abdominal distension and diffuse abdominal tenderness with guarding. Laboratory studies revealed an elevated leukocyte count with lymphocyte predominance, hyperkalemia, hyperphosphatemia, hyperuricemia and hypocalcemia, all concerning for tumor lysis syndrome (Table 1). According to the Cairo-Bishop classification of TLS, this patient's metabolic abnormalities and clinical signs fulfills the diagnosis of TLS (Table 2). Diagnosis requires two or more abnormal values for laboratory tumor lysis syndrome (i.e., hyperphosphatemia, hyperkalemia, hypocalcemia) along with clinical signs such as hypotension and acute kidney injury (creatinine value >1.5 times the upper limit of normal). 

**Table 1:** Laboratory values for day 1, day 2 and readmission day show marked hyperphosphatemia, hyperuricemia, hypocalcemia, and acute renal failure

Description	Day 1	Day 2	Readmission	Units	Reference range
	Value	Value	Value		
Leukocyte count	120.6	72.3	59.9	10^3/L	4.8-10.8
Hemoglobin	12.3	10.2	11.1	g/dL	13.5-17.0
Hematocrit	39.6	31.4	34.3	%	42-52
Mean Corpuscular Volume	92.5	91.4	91.6	fL	80.0-100.0
Platelets	284	15.4	195	10^3/uL	125-400
Neutrophils	23	25	7	%	36-66
Lymphocytes	73	68	89	16.0-43.5%	16.0-43.5
Monocytes	3	2	2	%	4.2-12.5
Sodium	122	127	130	mmol/L	136-145
Potassium	6.9	5.6	5.6	mmol/L	3.5-5.1
Chloride	84	88	97	mmol/L	98-107
Carbon dioxide	12	14	16	mmol/L	21-32
Anion gap	26	25	17	mmol/L	24-32
Glucose	146	142	107	mg/dL	65-99
Uric acid	9.4	16.5	9.7	mg/dl	2.1-8.0
Calcium	8.5	7.7	8.8	μmol/L	62-106
Phosphorus	12.2	9.1	7.7	mg/dl	2.7-4.5
Blood urea nitrogen	202	186	111	mg/dL	10.0-20.0
Creatinine	14.1	12.9	5.8	mg/dL	0.7-1.3

**Table 2:** The Cairo-Bishop diagnostic criteria for tumor lysis syndrome [12]

Laboratory criteria	
Uric acid	x ≥8.0 mg/dl or 25% increase from baseline
Potassium	x ≥ 6.0 mmol/l or 25% increase from baseline
Phosphorous	x ≥4.5 mg/dl (adults) or 25% increase from baseline
Calcium	x ≤ 1.75 mmol/l or 25% decrease from baseline
Clinical criteria	
(1) Creatinine: x ≥ 1.5 times upper limit of normal (age >12 years or age adjusted)	
(2) Cardiac arrhythmia/sudden death	
(3) Seizure	

**Figure 1:**
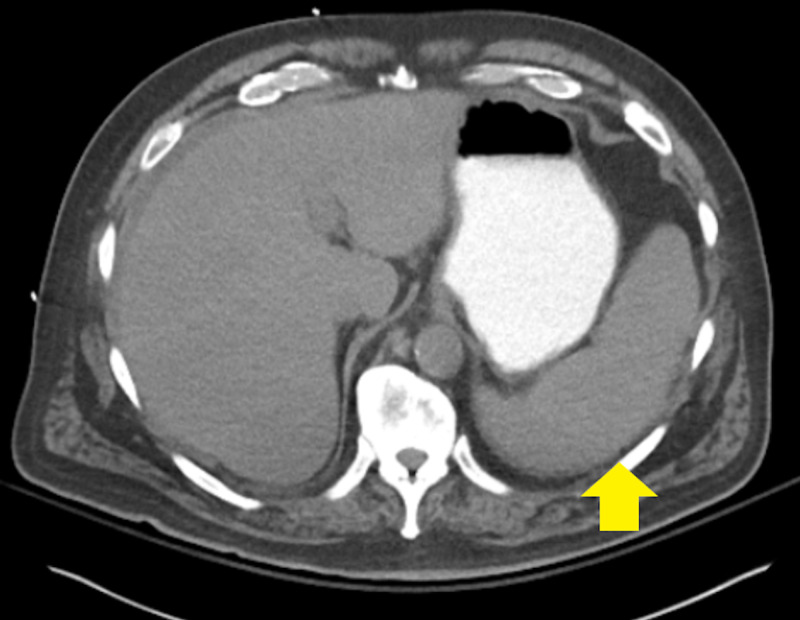
Computed tomography of the abdomen revealed mild splenomegaly in the patient, a common finding in chronic lymphocytic leukemia

EKG showed peaked T waves but was otherwise normal. Computed tomography (CT) scan of the abdomen and pelvis showed mild perihepatic ascites and splenomegaly (Figure 1). Ultrasound of retroperitoneum and kidneys showed no evidence of hydronephrosis. 

**Figure 2:**
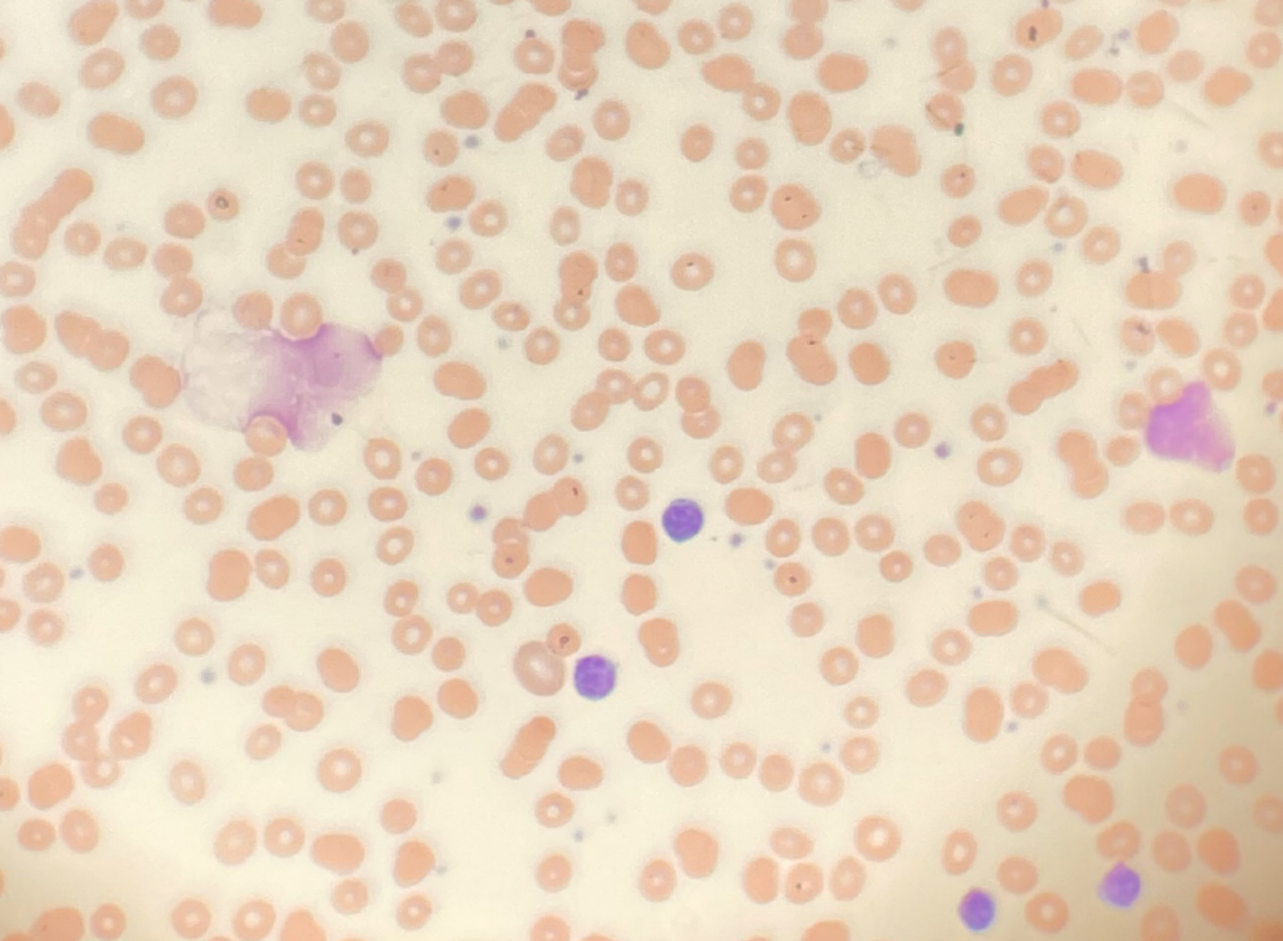
Peripheral smear performed on the patient shows prominent smudge cells (light pink, wide cells)

The patient was aggressively hydrated and administered rasburicase. Calcium gluconate and albuterol were also administered as temporizing measures for hyperkalemia. Four hours into the hospital stay, he went into hypovolemic shock, despite aggressive hydration, with mean arterial pressures plummeting into the 40’s. His blood pressure dropped to 80/44, pulse 93, respiratory rate 20, oxygen saturation-96% with 4 liters on nasal cannula. Pressors were administered through a central line, which stabilized him. On day 2 of admission, laboratory values showed improvement (Table 1). Histological analysis under the microscope revealed large mature lymphocytes with smudge cells (Figure 2). Flow cytometry confirmed the presence of an abnormal B-cell population consistent with B-cell chronic lymphocytic leukemia, with the following markers: CD19+, CD20+, CD23+, CD5+, CD10-. Flow cytometry results in combination with the metabolic derangements seen on admission, chiefly hyperkalemia, hyperphosphatemia, and hyperuricemia, suggested STLS. Fluid hydration was continued until the metabolic derangements were resolved and eventual normalization of kidney function. He was discharged with Foley and advised outpatient Hematology follow-up for further treatment and evaluation. Within two weeks, he was readmitted from a nursing home with signs of hypotension, acute kidney injury and abnormal labs such as hyperkalemia, hyperphosphatemia, and hyperuricemia, similar to his first admission (Table 1). Once again, his presenting laboratory values and clinical signs fulfilled the diagnosis of TLS. The patient was aggressively hydrated and received allopurinol and rasburicase. Fluorescence in situ hybridization (FISH) analysis revealed a complex cytogenetic CLL with unmutated IgVH indicating poor prognosis and indication for targeted therapy. He was diagnosed with Rai stage III CLL and started on ibrutinib, a tyrosine kinase inhibitor, 420 mg once daily. 

## Discussion

TLS describes the phenomenon of metabolic derangements that occurs following the initiation of cytotoxic chemotherapy in cancer patients. The rapid lysis of cells releases electrolytes, proteins, and nucleic acids causing serious downstream effects like acute renal failure and seizures [13,14]. The pathophysiology of TLS-mediated damage involves crystal precipitation in tissues that can cause fatal effects, like cardiac arrhythmias [15]. 

Intrinsic tumor qualities that increase the risk of TLS include rapid proliferative rate of the cancer, bulky tumors defined as greater than 10 cm in diameter or a white blood cell count greater than 50,000, involvement of bone marrow or other organs, and high chemosensitivity [15]. Certain clinical features also confer the risk of TLS. Pre-existing nephropathy, dehydration, oliguria, pretreatment hyperuricemia and/or hyperphosphatemia, and acidic urine all increase the probability of TLS [16,17].

Although TLS is appreciated more in hematological cancers, the prevalence varies greatly depending on the specific neoplasm. TLS is more often associated with acute leukemias and high-grade non-Hodgkin’s lymphoma, but is rare in chronic myeloid leukemia (CML), multiple myeloma, and CLL [17,18]. Of note, most solid tumors are low-risk for TLS. STLS has also been well-documented in hematological malignancies. Like TLS, acute leukemias and non-Hodgkin’s lymphoma have higher rates of STLS; however, it is exceedingly rare in the setting of CLL [17-19]. Moreso, a case of STLS in proven CLL has only been documented once, making this case extraordinary [20]. 

TLS prophylaxis recommendations are based on TLS risk [16]. Risk is classified as low-risk disease (LRD), intermediate-risk disease (IRD), and high-risk disease (HRD). LRD includes most solid tumors, chronic myeloid leukemia, Hodgkin's lymphoma, and CLL with a WBC <50 x 10^9^/L treated only with alkylating agents, among others. IRD notably includes highly chemotherapy-sensitive solid tumors (i.e. neuroblastoma, germ cell tumor, small-cell lung cancer) with bulky or advanced stage disease; also, CLL treated with fludarabine, rituximab, or lenalidomide, or venetoclax and lymph node ≥5 cm or absolute lymphocyte count ≥25 x 10^9^/L, and/or those with high WBC ≥50 x 10^9^/L are grouped in IRD. HRD involves CLL treated with venetoclax and lymph node ≥10 cm, or lymph node ≥5 cm and absolute lymphocyte count ≥25 x 10^9^/L and elevated baseline uric acid, Burkitt lymphoma and other highly aggressive neoplasms. Close monitoring of vitals and urine output is recommended for all three classes, as well as hydration. Administration of IV fluids is thought to help perfuse the kidney and improve glomerular filtration rate to minimize crystal nephropathy. Special attention should be given to patients in acute kidney injury or congestive heart failure, as volume overload becomes a concern in these patients. Also, it is recommended that all reversible forms of obstructive uropathy (i.e. BPH) be corrected so as to improve the success rate of prophylaxis treatment. Allopurinol, a hypoxanthine analog that competitively inhibits xanthine oxidase, is recommended in the setting of IRD, as long as uric acid is not ≥8 mg/dl. If uric acid is ≥8 mg/dl, or the patient falls under HRD, then rasburicase, a recombinant urate oxidase enzyme that catalyzes uric acid to water-soluble allantoin, if indicated as the prophylaxis treatment of choice. In patients with a history of glucose-6-phosphate dehydrogenase deficiency, rasburicase is contraindicated and is substituted with allopurinol instead.

Perhaps the most interesting aspect of this case was the inquiry of the main cause of kidney injury. Original thought revolved around the idea that the acute kidney injury was due to a combination of obstructive uropathy secondary to BPH and CLL spontaneous tumor lysis; however, the readmission case presented with similar abnormalities like acute kidney injury, hyperkalemia, hyperphosphatemia and hyperuricemia except for this time the patient was on Foley catheter. This proved the kidney injury was predominantly triggered by recurrent spontaneous tumor lysis secondary to the CLL.

## Conclusions

In this unusual patient case, spontaneous tumor lysis in the setting of pre-diagnosed CLL presented with oliguria, hypotension and severe acute renal failure, likely due to the precipitation of uric acid and calcium phosphate crystals in the tubules. Initially, there was confusion regarding the mechanism of acute kidney injury in this patient. Anuria, abdominal distention, and a history of BPH led us to suspect an obstructive uropathy exacerbating or triggering TLS. Subsequent imaging revealed no evidence of hydronephrosis, thus undermining the BPH etiology of kidney injury, and supporting nephropathy secondary to TLS. Furthermore, this patient was readmitted for a similar presentation even though the patient was catheterized, supporting the thesis of kidney damage secondary to cell lysis. Sequelae of TLS include seizures, arrhythmias, and acute kidney injury, with anuria presenting as an early warning sign. Undiagnosed and untreated, the mortality rate for TLS is approximately 15%. The risk factors, knowledge of the prevalence/outcomes and biochemical abnormalities associated with this potentially fatal syndrome must be recognized quickly by physicians to avoid fatal complications.
